# Effect of Coupling Medium on Penetration Depth in Microwave Medical Imaging

**DOI:** 10.3390/diagnostics12122906

**Published:** 2022-11-22

**Authors:** Wenyi Shao, Beibei Zhou

**Affiliations:** 1 The Russell H. Morgan Department of Radiology and Radiational Science, Johns Hopkins University School of Medicine, Baltimore, MD 21287, USA; 2 EMAI Inc., Laurel, MD 20723, USA

**Keywords:** microwave medical imaging, dielectric parameter, coupling liquid

## Abstract

In microwave medical imaging, the human skin reflects most of microwave energy due to the impedance mismatch between the air and the body. As a result, only a small portion of the microwave energy can enter the body and work for medical purpose. One solution to tackle this issue is to use a coupling (or matching) medium, which can reduce unwanted reflections on the skin and meanwhile improve spatial imaging resolution. A few types of fluids were measured in this paper for their dielectric properties between 500 MHz and 13.5 GHz. Measurements were performed by a Keysight programmable network analyzer (PNA) with a dielectric probe kit, and dielectric constant and conductivity of the fluids were presented in this paper. Then, quantitative computations were exercised to present the attenuations due to the reflection on the skin and to the loss in each coupling medium, based on the measured liquid dielectric values. Finally, electromagnetic simulations verified that the coupling liquid can allow more microwave energy to enter the body to allow for a more efficient medical examination.

## 1. Introduction

Microwave imaging is promising as a complementary medical imaging tool for breast tumor [1,2,3,4,5], brain stroke [6,7], and knee osteophyte detection [8]. It exploits difference in the electrical properties of various tissues at microwave frequencies to create an image. The main advantage of this method is that a microwave is a non-ionizing radiation, which means that it is much safer than the ionizing approaches that are currently used in clinics. The second advantage is the cost of a microwave medical imaging system is inexpensive; thus, it is affordable by middle-scale hospitals and small clinics. Many microwave detection prototypes have been invented [9,10,11,12,13,14] over the past few years. Encouraging results such as correctly localizing the lesions have been achieved.

Currently, a main challenge of this technique is the impedance mismatch between the human body (mainly the skin) and the air because the dielectric property of human tissues is significantly different from the air (dielectric constant 1.0006). This means that the human skin acts like a mirror reflecting most of the microwave energy. As a result, only a small portion of the radiated power can enter the body and work for medical purposes. To tackle this issue, two strategies have been proposed to improve the radiation efficiency. The first kind uses a skin-touching antenna, where the antenna is specifically designed to match the skin’s impedance [15]. During the examination, the antenna must touch tightly to the skin. However, the fabrication of such a skin-touching antenna is challenging. Any air gap between the skin and antenna will cause an impedance mismatch. The second strategy is to use a coupling medium, where both the antennas and the organ under test are immersed in a coupling liquid during the exam [14], e.g., a patient lying with back of head in the liquid for a brain imaging, or a woman patient in a prone position with her breast in the liquid for a breast exam. Since liquids have a larger dielectric constant than the air, the mismatch is thus reduced. Hence, the antenna needs to be designed to work in a specific liquid only (no need to touch tightly to the skin), so the design and fabrication of antennas are technically easier. In addition, a relatively large dielectric constant also benefits the reduction in the antenna’s physics dimension, which is desirable in microwave medical imaging. Last but not least, it has been reported that coupling liquid can also help improve spatial imaging resolution, due to an increased effective synthetic aperture of the antenna array [16,17]. As such, using coupling liquid is a favorite in many existing prototypes [11,12].

An ideal coupling medium for microwave medical imaging should have a relatively large dielectric constant (real part of a complex permittivity) because the skin’s dielectric constant is large (approximately 42 to 27 between 1 and 10 GHz [18]), and in the meantime the medium must be small lossy such that the microwave energy consumed in the liquid is insignificant. Pioneers have considered using water because of its large dielectric constant (approximately 79–61 in 1–10 GHz) [19,20,21]. However, water is quite lossy at microwave frequencies (effective conductivity from 0.27 to 17.6 S/m in 1–10 GHz, basically the larger the conductivity the more lossy the medium). Thus, water is not a preferred choice. Canola oil has also been used as a coupling liquid [22] because of its small conductivity (0.01 to 0.04 S/m from 1 to 10 GHz). However, the dielectric constant of canola oil is approximately 2.55 to 2.35 in the same frequency band, much smaller than the skin’s—just a little larger than the air. The most recent exploration of coupling fluids for microwave medical purpose is [23], in which the dielectric constant and conductivity of several kinds of liquid (vegetable oil, lemon extract, milk, etc., and their mixture) were measured. However, lemon and milk are both high water content media, and as a result the conductivity of all mixtures was thus large (larger than 0.5 S/m at 3 GHz for any mixture ratio). Moreover, oils and water are usually layered after they are mixed for a few minutes, and such deficiency was also mentioned in [23]. In addition, the measurements in [23] were limited up to a 3 GHz frequency, which is not high enough for some typical microwave medical applications such as breast examinations and knee examinations.

In the present article, the dielectric properties of multiple types of liquid that can be easily acquired from the shelf were measured from 500 MHz to 13.5 GHz. Then a further study concentrated on the two most feasible candidates: isopropyl 99% (1% water in volume) and glycerin. Lab measurement was performed by a Keysight programable vector network (PNA) with a dielectric probe kit. The open-ended coaxial probe method was applied in all measurements, and the built-in dielectric probe software in PNA calculated the complex permittivity of the liquid according to the probe signal. Data were then processed to plot the effective conductivity and dielectric constant (the latter is the real part of complex permittivity so no need of extra process) of the liquid. Our measurement confirms that the 99% isopropyl and glycerin are both good coupling mediums for microwave medical imaging. Rather than a mixture of glycerin and water (intensively studied in [24]), pure glycerin is of interest in this article, because even low water content could dramatically increase the conductivity (over 1.0 S/m in 1–3 GHz for glycerin concentrations in the range from 70 to 90% and the plane wave attenuation in the 90% glycerin is approximately 6 dB/cm [24]). Moreover, a single liquid is easy for use in practice, and avoids the issue of unstable performance due to an inaccurate ratio between multiple liquids. Based on the data acquired from the measurements, we then calculated the signal attenuation due to the loss in the coupling medium and the reflection from the skin. Finally, electromagnetic simulations were used to show the effect of isopropyl and glycerin on the field penetration in human-organ phantoms and were compared with the fields when no coupling liquid was adopted (phantoms exposed to air). Comparisons demonstrated that the coupling liquid could allow more electromagnetic energy to enter the body for a better microwave medical detection.

## 2. Open-Ended Coaxial Probe Method

**Figure 1:**
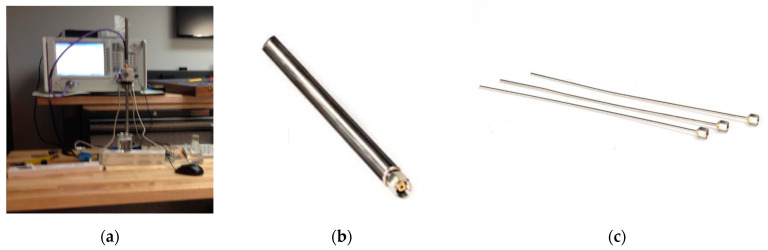
(**a**) Liquid dielectric measurement setup, (**b**) performance probe, and (**c**) slim-form probes.

The 85070E dielectric probe kit adopted in our measurement includes two types of probes. A slim probe is usually the best for liquids and soft semi-solids at room temperature, while a performance probe works fairly well for liquids but more typically for relatively high temperature. Figure 1a shows a measurement setup by the performance probe connected to a PNA in our lab, and (b) and (c) are the performance probe and three slim probes in the kit, respectively. Although both kinds of probes can work from a frequency of 500 MHz to 26 GHz, our PNA is limited by 13.5 GHz. Hence, all measurements were capped at 13.5 GHz, but that is sufficient for most microwave medical applications (e.g., the microwave brain imaging typically uses 1–2 GHz, and the breast imaging typically uses 2–10 GHz).

During the measurement, liquids under test were slowly added into a beaker to avoid air bubbles, then a probe was inserted into the liquid. The measurement started when it was confirmed there were no bubbles in the beaker. To guarantee the precision, a suitable amount of liquid must be used such that the probe’s bottom surface was more than 1 cm under the liquid surface and meanwhile at least 5 cm away from the beaker bottom. The latter prevents the reflection from the beaker bottom, which might confuse the probe. We used a high-performance flexible cable (valid up to 50 GHz) to connect the probe and the PNA, and 2.4 mm female SMA connectors to connect the cable and the probes.

Calibration must be conducted before the measurement, which virtually eliminates cable instability and system drift errors. We used the electronic calibration (ECal) module to perform the calibration. It includes three standard procedures: open, short, and water, performed at the end of the probe. The “open” is basically a measurement when the probe is exposed to air. The second procedure is a short-circuit measurement with a small accessory (slim-form probe and performance probe use different accessory) in the probe kit. The last one is a dielectric measurement of distilled water at room temperature. The calibration data are then transferred to the ECal module. The ECal module remains in line and a complete ECal calibration will be automatically performed before each measurement. Errors due to test port cable movement and system drift are thus removed by the calibration data.

## 3. Results

### 3.1. Measured Dielectric Values

**Figure 2:**
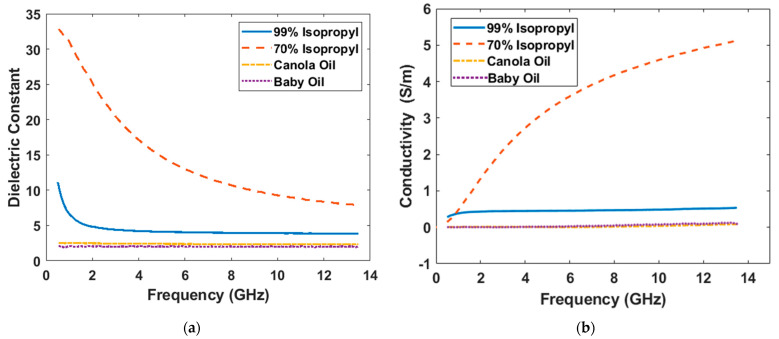
Measured liquid property data (20 °C). (**a**) dielectric constant, and (**b**) effective conductivity.

Firstly, four types of liquids were measured using the setup in Figure 1a: 99% isopropyl, 70% isopropyl, baby oil (main ingredient is mineral oil), and canola oil. The 70% isopropyl (30% water in volume) contains much water content so intuitively it might not be an ideal choice, but since it is widely used for medical purposes, it is selected here for comparisons. During the measurement, the PNA recorded the S11 parameter and then the 85070E dielectric probe software converted the data to permittivity values (real part and imaginary part separately). In Figure 2, the dielectric constant (real part of permittivity) and the effective conductivity values (manually converted from the imaginary part of permittivity) of the four fluids, canola oil, 70% isopropyl, 99% isopropyl, and baby oil over the 500 MHz to 13.5 GHz spectrum, are presented.

As expected, the dielectric constant and conductivity of 70% isopropyl are much higher than the other three, because of high water content. Technically, the dielectric constant of 70% isopropyl is the closest to skin, which creates the smallest reflection from the skin. However, the large conductivity prevents it from being an ideal coupling medium for many applications that require high-frequency signals (e.g., larger than 2 GHz). The dielectric data of canola oil were similar to what have been reported in [22]. Despite the desirable small conductivity, the dielectric constant of canola oil was too small; thus, canola oil is not a preferred coupling liquid either, the same as baby oil. Generally, the 99% isopropyl is the one best balance of the dielectric constant and effective conductivity among the four. A slim probe was employed for all four measurements.

**Figure 3:**
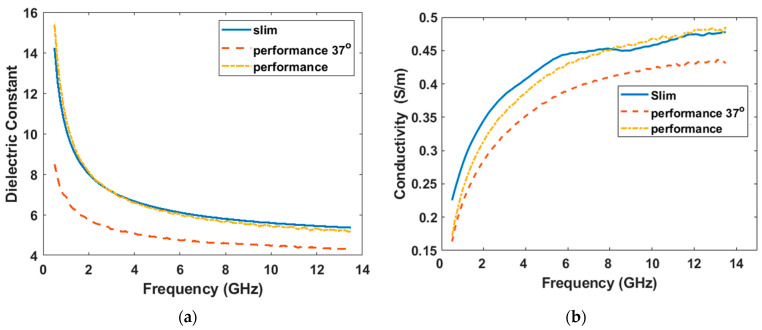
Glycerin measured by a slim-form probe and a performance probe at room temperature, and by a performance probe at 37 °C. (**a**) shows the dielectric constant. (**b**) shows the conductivity.

Figure 3 shows the measured dielectric data of glycerin. Firstly, the slim-form probe and the performance probe were used for the measurements at room temperature (20 °C). The results show that the data collected by the two probes are basically consistent. The dielectric constant of glycerin was larger than that of the 99% isopropyl, and the conductivity was smaller than that of 99% isopropyl, which indicates glycerin is even a better choice than 99% isopropyl. Next, for a more comfortable feeling in medical exams, fluids can be heated to body temperature. Therefore, we heated the same beaker of glycerin to 40 °C and waited until it cooled down to 37 °C, then a second measurement was performed (by using the performance probe due to increased temperature). Data show that as the temperature increases, both the dielectric constant and the conductivity decrease. For a better comparison, Table 1 presents the dielectric parameters of 99% isopropyl and glycerin between 1 and 10 GHz. It shows that the conductivity of two fluids is both smaller than 0.5 S/m even at 10 GHz, which is usually the up-limit frequency for many microwave medical exams. Considering the rapid evaporation rate of isopropyl alcohols, we remeasured the 99% isopropyl after it was placed in the breaker and exposed to air continuously for 15 min, which is sufficient for a complete microwave examination such as a breast or brain imaging [7,13,25]. However, no significant changes were observed in dielectric constant or conductivity, as shown in brackets in Table 1.

### 3.2. Quantitative Computation of the Attenuations

The data collected were then utilized to calculate the signal attenuation in each liquid while serving as the coupling medium. There are two sources of attenuation counted in this section: the loss in the liquid due to the lossy nature of the medium and the bounce back from the skin. We considered a plane wave illumination on the skin by a normal incidence. Calculation was based on the Fresnel’s equation as the plane wave had a normal incidence on a conducting surface, wherein the loss due to the reflection on skin was computed by

$$Loss_{ref}=20log\left|\frac{\tilde{E_{R}}}{\tilde{E_{I}}}\right|=20log\left|\frac{1-\tilde{\beta }}{1+\tilde{\beta }}\right|$$

where

$$\tilde{\beta }=\frac{v_{1}}{\omega }\tilde{k}$$

is a complex number as the skin must be considered as a conductor. $\tilde{k}$ is the complex wave number in the skin, $v_{1}$ is the phase velocity in the matching liquid, and ω is the angular frequency. $\tilde{E_{R}}$ standards for the reflected wave and $\tilde{E_{I}}$ denotes the incident wave, both in complex form.

**Figure 4:**
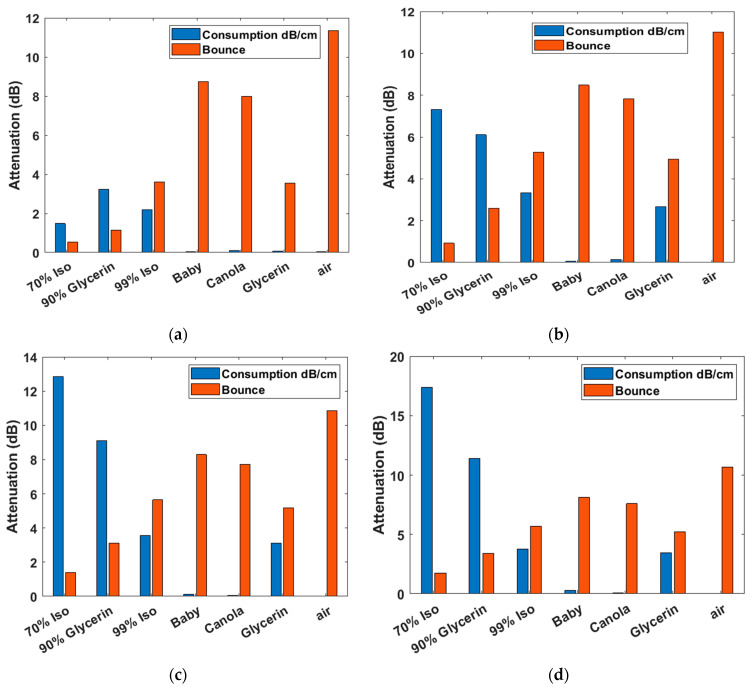
Plane wave attention due to consumption (dB/cm) in the lossy liquid shown in blue bars, and to the bounce back from skin shown in red bars, for the frequency (**a**) 1 GHz, (**b**) 3 GHz, (**c**) 5 GHz, and (**d**) 7 GHz.

The performance of each liquid at 1 GHz, 3 GHz, 5 GHz and 7 GHz is presented in Figure 4. The blue bars stand for the loss in the liquid, and the orange bars stand for the attenuation due to the reflection. For comparison, we also added the data of air and the 90% glycerin (10% water), where the raw dielectric data of 90% glycerin is derived from [24].

In practical medical examinations, antennas are usually placed one to several centimeters away from the skin. As expected, the signal attenuation due to the bounce between the skin and air, baby oil, or canola oil was large for all frequencies, but the values decrease to or less than 5 dB for all frequencies when glycerin, 90% glycerin, or 70% isopropyl was used. However, the attenuation due to the propagation loss (consumption) in 90% glycerin and 70% isopropyl increases rapidly with the frequency. If the water content is more than 10% in the water–glycerin mixture, the propagation loss will be even larger. This is the reason we prefer the 100% glycerin for wide-band microwave medical detections. In addition, it was interesting that both of the two kinds of attenuation for the 70% isopropyl were small at 1 GHz. This implies that for low-frequency applications (e.g., the microwave brain imaging), 70% isopropyl would be a suitable selection. For applications requiring high frequencies, glycerin would be the best. Note that analysis in this section did not consider the signal decay due to the field spread over range when a real antenna is adopted. Therefore, in the next section, simulations will be used to investigate field attenuations in dielectric medical phantoms.

### 3.3. Electromagnetic Simulations

**Figure 5:**
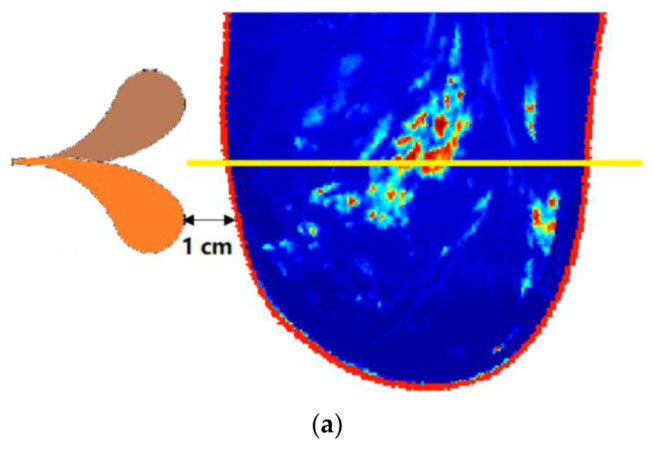

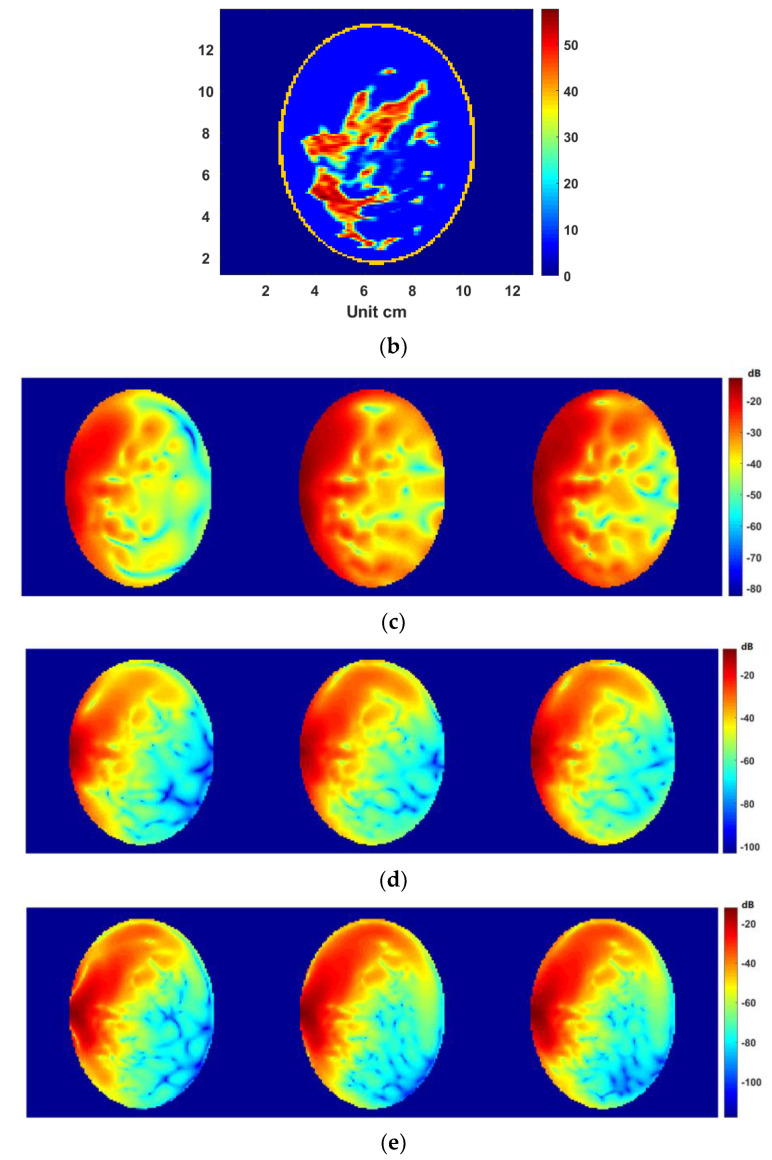
Electromagnetic field in a breast. (**a**) Setup of the measurement; (**b**) an extracted slice from the 3D breast model in the trans−axial plane. (**c**) From the left to right: 3 GHz field in the breast when no liquid was used (in air), field in the breast when 99% isopropyl was used, and field in the breast when glycerin was used, respectively. (**d**) From the left to right: 5 GHz field in the breast when no liquid was used (in air), field in the breast when 99% isopropyl was used, and field in the breast when glycerin was used, respectively. (**e**) From the left to right: 7 GHz field in the breast when no liquid was used (in air), field in the breast when 99% isopropyl was used, and field in the breast when glycerin was used, respectively.

Simulations were conducted to observe the contribution of coupling liquids to microwave medical detection. The first test was a 3D breast model illuminated by an antipodal Vivaldi antenna [26] which was specifically designed to work in air, 99% isopropyl, or glycerin. The 3D dielectric breast model was derived from a magnetic resonance imaging (MRI) breast model and then converted to dielectric data by the University of Wisconsin electromagnetic research group [27]. Figure 5a shows the measurement setup. The patient was in a prone position with her breast hanging down in a tank. The 3D breast model is composed of 128×128×128 voxels. The voxel size is 1 mm along all three dimensions. In this project, voxels out of the breast region were assigned dielectric parameters of the coupling liquid. Since the antenna is supposed to be placed to the left of the breast, the entire simulation model was thus extended to 160×128×128 voxels to accommodate the antenna (to the left). Note that the real physical dimension of the antenna working in air is larger than the ones working in liquids (so the simulation model was expanded to 192×128×128 to accommodate the large antenna in air), and the antenna designed for the 99% isopropyl was also slightly different from the one for in glycerin. Antenna polarization was along the vertical direction. Figure 5b shows the dielectric constant (at 3 GHz) of the breast in a trans-axial plane, which corresponds to the antenna gap central (shown by a yellow line in Figure 5a). When the tank is filled with a certain liquid, both the antenna and the breast model were assumed to be immersed in such liquid at room temperature.

The simulation was based on a frequency-dependence finite difference time-domain method [28]. The time-domain data were then converted to frequency domain by a Fourier transform, and then the data at a certain frequency were extracted. Figure 5c shows the magnitude of a 3 GHz electromagnetic field in the breast (the slice shown in Figure 5b) as the breast was exposed in air and immersed in 99% isopropyl and in glycerin, respectively. It was noticed that when no coupling medium was used, the field in the breast can be less than −80 dB in some positions. Thus, the scattered signal from these positions would hardly be received by a reception device located near the transmitter (or by a transceiver if a monostatic mode was adopted), due to the limited dynamic range of a reception device [29,30], such as a PNA. Performance was greatly improved when a coupling medium was employed. As the 99% isopropyl or glycerin was used, the minimum of the field increased to approximately −70 dB, which means the scattered fields by any position in the breast can be detected even though the receiver is placed to the left of the phantom (near the transmitter antenna) when a PNA with 140-dB dynamic range is offered.

Since higher frequencies than 3 GHz are often used in microwave breast exams, we also extracted the fields for 5 GHz and 7 GHz, which are presented in Figure 5d,e, respectively. At microwave frequencies, the dielectric-constant difference between the skin and the air (or coupling liquid) reduces as the frequency increases, indicating that higher-frequency microwave signals can more easily enter the organ. However, the conductivity (or the loss tangent) of human tissues increases with the frequency, meaning that high-frequency signals decay more rapidly in the organ tissues. Therefore, the fields for 5 GHz and 7 GHz turned out to be smaller than 3 GHz. Note that Figure 5c–e only present the electromagnetic field in the breast. The color out of the breast does not have physical meanings.

**Figure 6:**
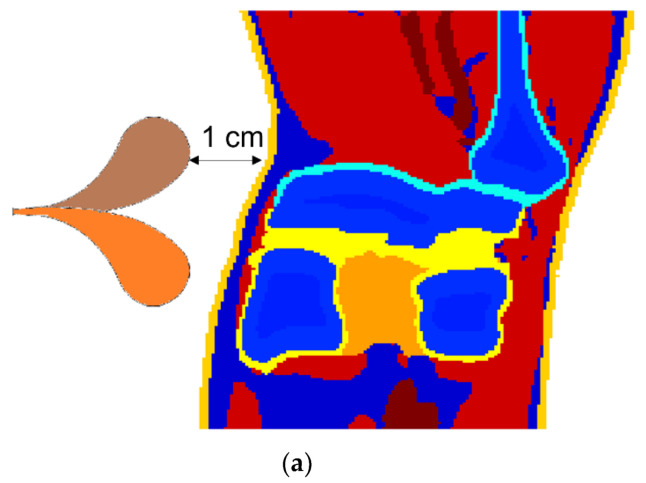

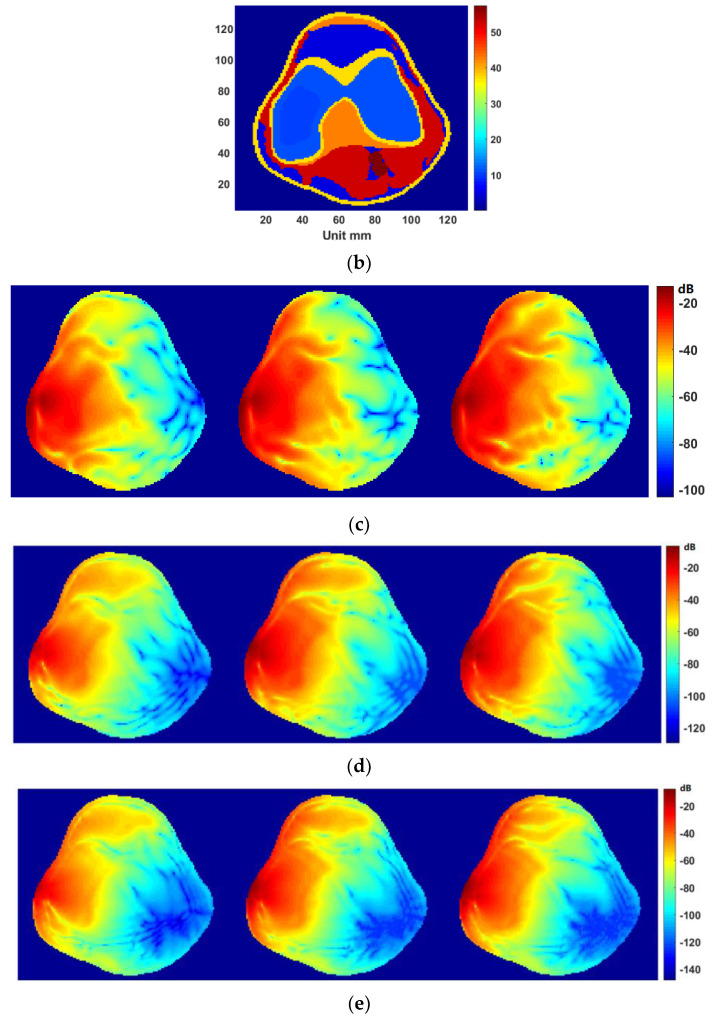
Electromagnetic field in a knee. (**a**) Measurement setup with an antipodal Vivaldi antenna. (**b**) a slice in the trans−axial plane extracted from the 3D knee model. (**c**) From left to right: 3 GHz field in the slice shown in (**b**) when the knee is exposed in air, 99% isopropyl, and in glycerin, respectively. (**d**) From left to right: 5 GHz field in the slice shown in (**b**) when the knee is exposed in air, 99% isopropyl, and in glycerin, respectively. (**e**) From left to right: 7 GHz field in the slice shown in (**b**) when the knee is exposed in air, 99% isopropyl, and in glycerin, respectively.

Figure 6 shows the electromagnetic field in a knee when it was illuminated by the same antennas in the breast detection in this paper, placed to the left of the knee (Figure 6a). The 3D dielectric knee model was truncated from a full human-body model [31] that consists of 45 types of tissues, wherein the knee model contains 8 types of tissues: bone cortical, cartilage, marrow, fat, blood, muscle, skin, and tendon. The 3D knee model is composed of 128×128×128 voxels but was expanded to 160×128×128 to accommodate the antenna on the left of the knee. Voxels out of the knee were air but were re-assigned the parameters of matching liquid when matching liquid was employed. The voxel size of the model was 1 mm in all three directions (x, y, and z). Figure 6b is a 2D slice extracted from the 3D knee model (dielectric map for 3 GHz). The antenna polarization is normal to the slice shown in Figure 6b. Figure 6c shows the fields in the knee when the knee and the antenna were exposed in air, immersed in 99% isopropyl, and in glycerin, respectively. It was noticed that the area of less than −70 dB was reduced when a coupling liquid was used. This verifies that the field penetration into the knee is improved if a coupling liquid is employed. For higher-frequency applications, Figure 6d,e presented the fields at 5 GHz and 7 GHz in the knee, respectively, when the knee was considered being exposed to air, in 99% isopropyl, or in glycerin.

**Figure 7:**
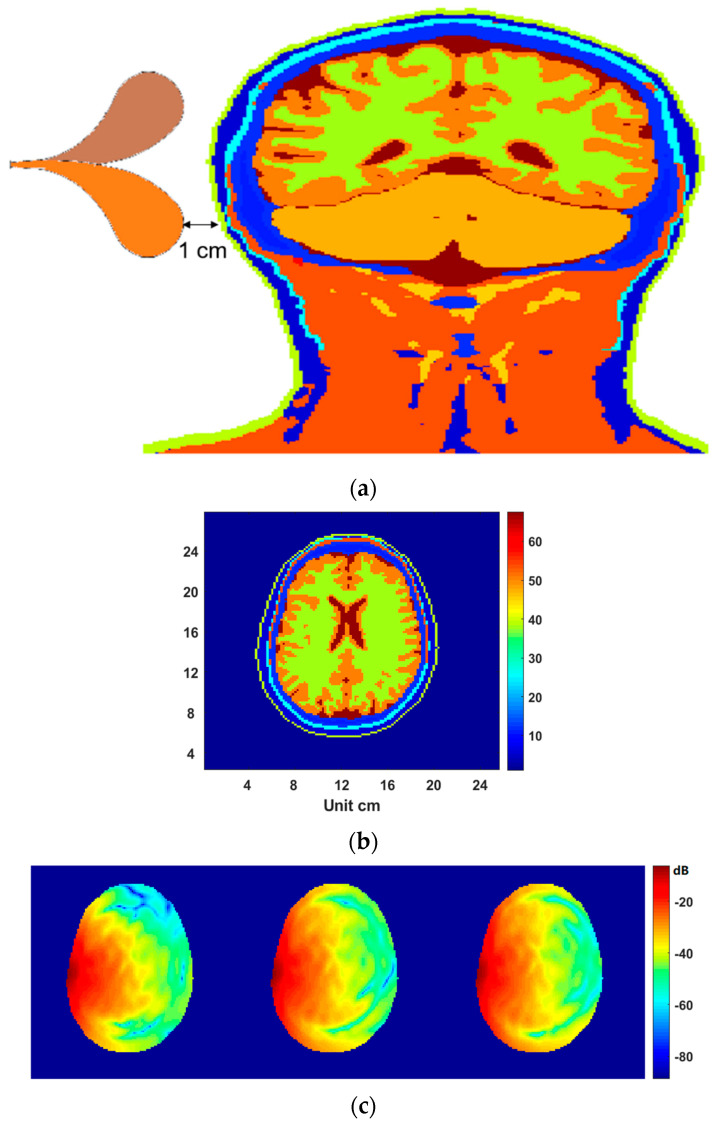
Electromagnetic field in a head. (**a**) measurement setup; (**b**) a slice extracted from the 3D dielectric head model in the trans−axial plane; (**c**) From left to right: field in the slice plane shown in (**b**) when the head is exposed in air, in 70% isopropyl, and in glycerin, respectively.

Another test is a simulation of a microwave head examination. Figure 7a shows a coronal slice of the head, in which plane the antenna was positioned. The head model is also derived from the full-body model [31] but with an isotropic voxel size of 2 mm. The dimension of the head model was 128×128×128, but was expanded to 160×128×128 to accommodate the antenna to the left (192×128×128 to accommodate the large antenna for working in the air). Note that the physical dimension of the antenna working in air is much larger than the ones designed for working in liquids. Since the head dimension is larger than the breasts and knees, and the tissues in head are more lossy to microwave signals, lower frequencies such as 1–2 GHz are often used in microwave head imaging [32]. Figure 7b shows a dielectric slice extracted from the 3D model for 1.5 GHz, which corresponds to the gap central of the antenna. The antenna polarization is normal to this slice. When the 3D simulation was complete, the field data in such a slice for 1.5 GHz were extracted out and presented in Figure 7c. Considering 70% isopropyl may have excellent performances for low-frequency applications, instead of the 99% isopropyl, the 70% isopropyl was used and compared with glycerin and air. When no coupling medium was used (in air), the minimum field value in the head was approximately −85 db. In contrast, the minimum increased to approximately −76 dB when the 70% isopropyl or glycerin was used for coupling. It should be noted that we also calculated the attenuation effect in 80% glycerin (20% water) and 70% glycerin (30% water) by using the reported dielectric data in [24]. For the 1.5 GHz frequency, although the attenuation due to the bounce on skin was basically equivalent to the 70% isopropyl, the loss in the 80% and 70% glycerin was higher than the loss in 70% isopropyl. Hence, the performance of the glycerin–water mixture was not further investigated in this work.

## 4. Conclusions

A coupling medium can reduce the impedance mismatch in microwave medical exams; thus, dielectric measurements for liquids have been investigated by a few researchers in order to find an ideal coupling liquid for specific medical applications. In this paper, we explored the effect of several coupling liquids on microwave medical imaging by quantitative computations and electromagnetic simulations, using the in-house measurement data. Results indicate that pure glycerin is a good choice for serving as the coupling medium for most microwave medical applications. But 70% isopropyl is suitable for low-frequency applications such as head imaging. A clue of this is that the conductivity of 70% isopropyl is comparable to the 99% isopropyl at low frequencies, which can be found in Figure 2b; meanwhile, its dielectric constant is much larger than the 99% isopropyl in Figure 2a. To introduce the coupling medium in detections, researchers who intended to use a virtual synthetic aperture array will need to consider a way to safely and efficiently move the antennas in a tank filled with liquid. From this point of view, a real antenna array comprising a plurality of antennas [33] will have advantages because no movement is needed for the antennas.

## Figures and Tables

**Table 1:** Comparison between 99% Isopropyl and Glycerin at room temperature by a slim probe.

Frequency (GHz)	99% Isopropyl (+15 min)		Glycerin	
	Dielectric Constant	Conductivity (S/m)	Dielectric Constant	Conductivity (S/m)
1	6.56 (6.61)	0.38 (0.39)	10.1	0.28
2	4.84 (4.86)	0.43 (0.43)	8.0	0.34
3	4.39 (4.40)	0.44 (0.44)	7.17	0.38
4	4.21 (4.21)	0.45 (0.45)	6.66	0.40
5	4.11 (4.13)	0.45 (0.45)	6.34	0.43
6	4.04 (4.05)	0.45 (0.46)	6.11	0.43
7	4.00 (4.00)	0.46 (0.46)	5.94	0.44
8	3.96 (3.96)	0.47 (0.47)	5.79	0.45
9	3.92 (3.93)	0.47 (0.48)	5.68	0.45
10	3.90 (3.91)	0.48 (0.48)	5.59	0.46

## Data Availability

Not applicable.
